# Cost of overweight, obesity, and related complications in Switzerland 2021

**DOI:** 10.3389/fpubh.2024.1335115

**Published:** 2024-07-12

**Authors:** David Steinl, Pascale Holzerny, Stephan Ruckdäschel, David Fäh, Zoltan Pataky, Ralph Peterli, Bernd Schultes, Susanne Landolt, Timo Pollak

**Affiliations:** ^1^HealthEcon AG, Basel, Switzerland; ^2^School of Health Professions, Bern University of Applied Sciences (BFH), Bern, Switzerland; ^3^Department of Epidemiology, Biostatistics and Prevention Institute, University of Zurich, Zurich, Switzerland; ^4^Unit of Therapeutic Patient Education, Division of Endocrinology, Diabetology, Nutrition and Therapeutic Patient Education, World Health Organization Collaborating Centre, Geneva University Hospitals and University of Geneva, Geneva, Switzerland; ^5^Clarunis, Department of Visceral Surgery, University Centre for Gastrointestinal and Liver Diseases, St. Clara Hospital and University Hospital Basel, Basel, Switzerland; ^6^Metabolic Center St. Gallen, friendlyDocs Ltd., St. Gallen, Switzerland; ^7^Novo Nordisk Pharma AG, Zürich, Switzerland; ^8^Novo Nordisk Denmark A/S, Copenhagen, Denmark

**Keywords:** overweight, obesity, epidemiology, treatment, healthcare costs, Switzerland, obesity-related complications, bariatric surgery

## Abstract

**Background:**

The prevalence of obesity has increased significantly in recent decades. Today, it is estimated that more than one-third of the world’s population has overweight or obesity, rendering it one of the most significant global health concerns. This article provides a current estimate of the direct costs associated with managing overweight and obesity, including treatment of related complications, among adolescents (≥15 years) and adults in Switzerland.

**Methods:**

Prevalence of overweight and obesity based on the BMI reported in the 2017 Swiss Health Survey was extrapolated to 2021. Systematic literature searches were performed to identify treatment costs and epidemiological data of obesity-related complications and costs were extrapolated to 2021. Costing methodology was based on available source data for individual related complications. Treatment costs for complications attributable to overweight and obesity were estimated by applying their population attributable fraction (PAF).

**Results:**

More than 3.1 million inhabitants of Switzerland aged ≥15 years met the criteria for overweight or obesity in 2021. The prevalence of overweight increase over the past decades from 30.4% in 1992 to 41.9% in 2017 while prevalence of obesity doubled from 5.4 to 11.3%. Overall, the total attributable costs of overweight and obesity caused by seven assessed obesity-related complications (asthma, coronary heart disease, depression, diabetes mellitus, hypertension, osteoarthritis, and stroke) are estimated at CHF 3657–5208 million with most of the costs (97–98%) caused by the assessed obesity-related complications. Only 2–3% of the total costs were attributable to the combined direct management of overweight and obesity by bariatric surgery (CHF 83 million), pharmacological therapy (CHF 26 million) and dietary counseling (CHF 18 million).

**Conclusion:**

Overweight and obesity impose a significant cost impact on the Swiss healthcare system, accounting for 4.2–6.1% of total healthcare expenditures in 2021. Notably, direct treatment of overweight and obesity accounts for only 0.08–0.18% of the total healthcare expenditures. The analysis also revealed a significant lack of available health economic evidence, necessitating the use of assumptions and approximations in this estimation. This is noteworthy, as respective data would be available in healthcare systems but are either unpublished or inaccessible.

## 1 Introduction

Obesity is a complex, multifactorial, and difficult-to-treat chronic disease that is associated with premature mortality and chronic morbidity such as diabetes, cardiovascular diseases, or malignancies, which may severely compromise patients’ life expectancy and their overall quality of life (1, 2). The impairment of activities of daily living and the perceived stigmatization contribute significantly to the burden, resulting in a self-perpetuating cycle that adversely impacts the individual’s health, as well as psychological and psychosocial functioning, further solidifying this vicious cycle (3, 4).

In addition to the adverse effects of overweight and obesity on the individual, these conditions also contribute to the development of several noncommunicable diseases from a public health perspective, leading to an increased consumption of healthcare resources with implications for healthcare systems and societies (5, 6). In response to the global rise in obesity prevalence, the World Health Organization (WHO) declared obesity an epidemic in 1997, citing the overwhelming consequences on personal health as well as public healthcare systems (7). The prevalence of obesity has increased significantly in recent decades, with estimates indicating that more than one-third of the world’s population has overweight or obesity, rendering it one of the most significant global health concerns (8).

The determination of the health economic costs of a disease is essential for determining preventive measures and decisions about allocation of healthcare budgets. Unfortunately, estimating the cost of obesity is challenging for this chronic condition, that can be thought of as a risk factor for other diseases with a complex health economic footprint. Published data regarding the cost of obesity is rare. Especially for Switzerland, there is only very limited data available with the most relevant publication by Schneider and Venetz in June 2014, which was commissioned by the Federal office of public health (FOPH) (9). The primary objective of the present study therefore was to update a previous cost estimate with most current economic and epidemiologic data. This update focuses on direct costs, i.e., the costs directly incurred by the management of overweight and obesity, as well as the treatment of the seven most health economically impactful obesity-related complications. Indirect costs typically including productivity losses, as well as presenteeism and absenteeism, although significant in their magnitude and a major cost driver for the chronic diseases presented here, play a less important role in public health decision making, although they represent a substantial economic burden. This is due to their inherent challenges of accurately quantifying such costs and the structural allocation of these costs to the social systems rather than healthcare funds, which is why this study focused on estimating of direct costs only.

The direct costs of managing and treating patients with overweight or obesity considered in this study include dietary counseling, pharmacologic therapy, and bariatric surgery, as well as treatment of obesity-related complications (inpatient and outpatient services as well as prescription medications). The obesity-related complications assessed were based on the highest costs attributable to overweight and obesity as reported by Schneider and Venetz in 2014 and include asthma, coronary heart disease (CHD), depression, diabetes mellitus, hypertension, osteoarthritis, and stroke (9). To estimate the costs of the selected obesity-related complications, it was necessary to determine the contribution of overweight and obesity to the occurrence of each disease. The population attributable fraction (PAF) expresses the extent to which a specific risk factor (or group of risk factors) contributes to the burden of a disease (i.e., the incidence of the disease and, if monetary values are assigned, the cost of the disease) (10).

## 2 Materials and methods

The Body Mass Index (BMI) is an internationally applied measure to classify individuals according to the relationship between their body height and weight and allows comparison of data between populations or longitudinally within a population (11). The range for (non-Asian) adults is defined by the WHO as underweight <18.5 kg/m^2^, normal weight 18.5–24.9 kg/m^2^, overweight (pre-obesity) 25.0–29.9 kg/m^2^ and obesity ≥30.0 kg/m^2^ (12). These definitions were used in the present analysis.

### 2.1 Prevalence of overweight and obesity Switzerland

Data from six Swiss Health Survey (SHS) conducted at 5-year intervals since 1992/93 were used to estimate the prevalence of overweight and obesity in adolescents aged 15 years and older and the adult Swiss population (permanent residents, regardless of citizenship status) (13). The adjustment of the population to 2021 was based on the 2017 SHS, the most recent version available at the time of the present study, with linear extrapolation for the Swiss population as reported by the Federal Statistical Office (FSO) for 31.12.2021 (14).

### 2.2 Dietary counseling costs

The number of dietary consultations was requested from Santésuisse for January to June 2022, as the COVID-19 pandemic led to a reduction in consultations in 2021 and would have underestimated the costs. The number of inpatient and outpatient consultations was requested, broken down by category of consultation (e.g., first, second to sixth) and the corresponding tax point values (15, 16). One tax point value was equal to one Swiss franc (CHF) at the time of this study (17, 18). Due to a lack of available data on underlying diagnoses for dietary counseling, expert interviews were conducted to inquire about the proportions of counseling due to obesity and overweight.

### 2.3 Pharmacology therapy costs

Swiss market data was available detailing anti-obesity medications (AOM) packages sold including orlistat and liraglutide for January to June 2022, to account for changes in market patterns following the initiation of reimbursement of liraglutide in 2020 for treatment of overweight and obesity in Switzerland (19).

### 2.4 Bariatric surgical therapy costs

The swissDRG Datenspiegel v.11 was systematically searched for all Swiss Classification of Procedures Classification (CHOP) codes designated by the Swiss Society for the Study of morbid Obesity and metabolic Disorders (SMOB) for bariatric surgeries in 2019 (20, 21). Information was extracted including number of performed procedures per diagnosis related group (DRG) and average DRG prices. Classification and allocation to a DRG was based on treatment type and complexity. Bariatric surgery costs were estimated based on the number of procedures performed in 2019, as the COVID-19 pandemic led to a reduction in surgical procedures performed in 2021 and would have underestimated the costs. Data for 2022 were not available at the time of preparation.

### 2.5 Cost of selected overweight and obesity-related complications

Selection of obesity-related complications was based on the highest cost attributable to overweight and obesity in 2012 and included asthma, CHD, depression, diabetes mellitus, hypertension, osteoarthritis and stroke (9). Cost of illness were multiplied with the specific PAF for each disease to estimate the fraction of cost that are attributable to overweight and obesity.

To identify respective cost data as well as data for PAF estimation, two systematic literature searches were performed. The search strategy is detailed in Supplementary Tables S8, S9.

1. Systematic literature search to identify costs of selected obesity-related complications: MEDLINE, guideline search portals (AWMF, G-I-N), selected medical societies (SMOB, SGE, SGES, SGED,) and Swiss bodies of interest (BAG, BFS, Obsan, Swiss Medical Board)
2. Systematic literature search to identify relative risks (RR), odds ratios (OR) or PAF of selected obesity-related complications regarding the BMI: MEDLINE

In accordance with established protocols, duplicates within the search results were removed, and a title and abstract screening was performed to identify relevant publications, which were then subjected to a comprehensive full-text assessment. Publications were included for analysis if they addressed the research question and provided Swiss data. If neither Swiss nor data for countries with a comparable socio-economic structure (preference given to neighboring countries with similar health systems, e.g., Germany) were available, the best available evidence was applied under consideration of data validity as well as comparability to Switzerland. Subsequently, additional cost data from Germany, France and the Netherlands and reported data for RRs, ORs or PAFs based on analyses of German, French, UK, USA, Swedish, Finnish datasets as well as a European meta-analysis were used and discussed in the result section of this publication.

### 2.6 Extrapolation of costs for Switzerland 2021

Relevant costs of identified publications were converted to Swiss Francs (CHF) in a two-step method involving conversion and extrapolation. First, all costs data of foreign currency were converted to CHF by applying the Purchasing Power Parity (PPP) for that respective source year (22). In a second step, the cost data in CHF for that source year prices were extrapolated to 2021 price level based on the annual changes in the Consumer Price Index (CPI, “Landesindex der Konsumentenpreise”) (23).

An alternative extrapolation based on the annual changes in total healthcare expenditures was performed and is shown in the appendix for the purpose of comparison with Schneider and Venetz 2014 (see Supplementary Tables S6, S7) (24). However, the extrapolation based on CPI is preferred, as it is more sensitive for individual cost components and less prone to grossly overestimate cost data from older sources. After conversion to 2021 CHF price levels, costs were adjusted to the Swiss population of adolescents aged 15 years and older and adults in 2021 based on linear extrapolation from data reported by the FSO for 31.12.2021 (14).

### 2.7 Estimating the cost of selected complications attributable to overweight and obesity

The costs of treating overweight and obesity-related long-term complications were estimated using PAFs. Wherever possible, published RRs or ORs from two independent sources were used to calculate two PAFs to minimize bias and a range is presented (see Table 1). Disease-specific PAFs were calculated based on the RR (or OR) reported in the identified publication for both overweight and obesity according to the formula with (p) as the SHS 2017 prevalence of overweight and obesity, respectively, and the corresponding disease-specific RR (or respectively):


$$PAF=\frac{p\left(RR-1\right)}{p\left(RR-1\right)+1}$$

**Table 1:** Population attributable fractions (PAF) for Switzerland for selected diseases by overweight and obesity.

Disease	PAF overweight 25 ≤ BMI < 30	PAF obesity BMI ≥ 30	References
Asthma	6.2*	6.4*	Zemp Stutz et al. (10)
	20.3*	18.7*	Ma et al. (25)
CHD	14.1*	11.3*	Flint et al. (26)
	6.6	4.7	Liu et al. (27)
Depression	3.7*	5.4*	Luppino et al. (28)
Diabetes mellitus	16.2*	42.9*	Davin et al. (29)
	38.4*	41.4*	Zemp Stutz et al. (10)
Hypertension	18.1*	16.8*	Davin et al. (29)
	23.7*	24.9*	Guh et al. (30)
Osteoarthritis (hip)	13.3*	15.7*	Lohmander et al. (31)
	14.9*	14.4*	Holliday et al. (32)
Osteoarthritis (knee)	35.5	34.3	Lohmander et al. (31)
	23.8	24.7	Muthuri et al. (33)
Stroke	5.9*	5.4*	Zemp Stutz et al. (10)
	18.3*	17.4*	Winter et al. (34)

*Weighted mean to account for differences between males and females in the respective incidence/prevalence.

BMI, Body mass index; CHD, Coronary heart disease; PAF, Population attributable fraction.

For three diseases (asthma, diabetes mellitus and stroke), PAFs reported by Swiss TPH were directly applied (see Table 1) (10).

## 3 Results

### 3.1 Prevalence of overweight and obesity in Switzerland in 2021

**Figure 1:**
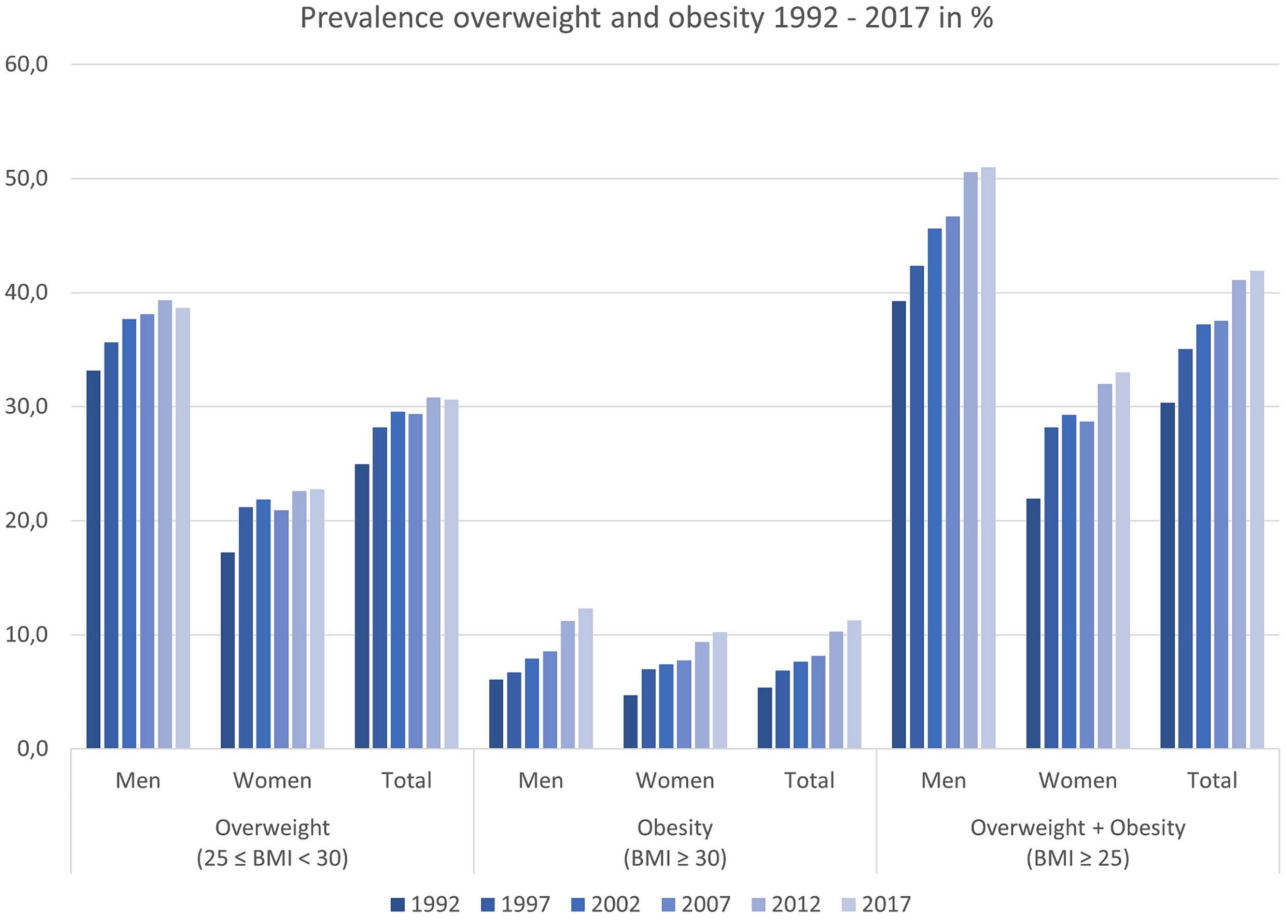
Overall development of overweight and obesity prevalence in Switzerland from 1992 to 2017 (Inhabitants ≥15 years of age) (13).

Over the past 25 years, the percentage of people living with overweight and obesity among adolescents aged 15 years and older and adults in Switzerland has increased significantly from 30.4% in 1992 to 41.9% in 2017 (see Figure 1). The observed prevalence of overweight was 25.0% in 1992 and 30.6% in 2017. Both sexes experienced an equal increase in the prevalence of overweight over time with men reporting a prevalence twice as high as women in absolute numbers. Specifically for obesity, the prevalence in Switzerland doubled from 5.4% in 1992 to 11.3% overall in 2017 (men: 6.1% to 12.3%; women: 4.7% to 10.2%), with the increase evenly distributed across all age groups.

Extrapolating from the 2017 SHS results, more than 3.1 million Swiss residents aged 15 years and older are considered having overweight or obesity based on the definition of BMI ≥ 25 kg/m^2^. The number of patients with overweight or obesity is higher in men (1.8 million) compared to women (1.3 million).

### 3.2 Direct cost of overweight and obesity

Treatment of overweight and obesity in Switzerland through lifestyle changes include a combination of approaches including dietary education, exercise, physiotherapy, and general behavioral therapies with weight-reducing drugs and/or bariatric surgery as a potential add-on therapy.

#### 3.2.1 Dietary counseling

Extrapolated data provided by Santésuisse for Switzerland in 2022 amounted to 429148 dietary consultations (see Supplementary Table S1). The experts consulted estimate that outpatient dietary consultations for overweight and obesity account about 80% of the reported outpatient consultations. For inpatient consultations, this rate is lower at about 20%, because inpatient consultations focus primarily on dietary therapy during hospitalization for conditions such as gastrointestinal surgery, cardiovascular events, renal disease, severe malnutrition, diabetes mellitus and others.

A total of 230,481 consultations attributable to overweight and obesity were estimated for 2022 (see Supplementary Table S1). Based on the currently valid tariff contracts, the total costs for dietary consultations in Switzerland for 2022 is estimated at CHF 18.3 million overall, with 84% of the costs attributable to outpatient dietary consultations (CHF 15.4 million) (15, 16).

#### 3.2.2 Pharmacological therapy

Anti-obesity medication (AOM) approved by Swissmedic for weight reduction include orlistat and liraglutide for adult patients presenting with obesity (BMI ≥30 kg/m^2^) or for patients presenting with overweight (orlistat: BMI ≥ 28 kg/m^2^; liraglutide: BMI ≥ 27 kg/m^2^) and at least one weight-related comorbidity (pre-diabetes or diabetes mellitus type 2, arterial hypertension, or dyslipidemia) (35, 36).

Based on confidential sales data for the first half of 2022, it is estimated that 184,461 packages of AOMs were sold, representing an estimated total sales volume of CHF 26.0 million in Switzerland in 2022 (37) (see Table 2). Notably, experts interviewed for this study indicated that a significant portion of this amount will not be covered by the Swiss mandatory health insurance (OKP) but will be paid out-of-pocket by the patients themselves.

#### 3.2.3 Bariatric surgical therapy

Of the 4926 bariatric surgeries performed in Switzerland 2019, the most common procedures were forms of gastric bypasses and sleeve resection, that together made up 73.7 and 20.4%, respectively (see Supplementary Tables S2, S3). Other bariatric procedures were available and included restrictive gastric procedures (e.g., implementation of an adjustable gastric band; pouch-forming in vertical banded gastroplasty), but are historically declining in total numbers of procedures in favor of the more effective procedures that are gastric bypasses and sleeve resections (38). Biliopancreatic diversion, although highly effective, is performed very rarely due to complexity of the procedure and negative long-term effects.

The total costs of bariatric surgeries in Switzerland in 2021 is estimated at CHF 82.6 Mio, based on the number of procedures per DRG and the average costs of each DRG in 2019, adjusted for population increase and inflation with an average costs of CHF 16,768 per procedure (see Table 2).

### 3.3 Costs of selected overweight and obesity-related complications

#### 3.3.1 Asthma

In 2017, overall 5.1% (4.6% in men and 5.6% in women) of the Swiss population ≥ 15 years of age stated they had asthma within the last 12 months (39). There are 377,567 people (166,245 men, 211,322 women) in Switzerland suffering from asthma in 2021 (14, 39). To estimate the costs for 2021, published Swiss representative data reported by Scuzs et al. was extrapolated (40). Using the described extrapolation methods for annual changes in CPI in healthcare and adjustment to the 2021 Swiss population, the resulting direct costs for asthma in Switzerland 2021 were CHF 574 million (including inpatient CHF 340.9 million, outpatient: CHF 77.4 million and prescribed medication CHF 155.6 million; see Supplementary Table S5).

PAFs for asthma due to overweight and obesity were calculated based on the risks reported by Ma et al. (US data 2005–2006) and the current overweight and obesity prevalence data from the SHS 2017, resulting in a PAF for overweight of 20.3% and a PAF for obesity of 18.7% (see Table 1) (13, 25). To consider the uncertainty, a second data source of adjusted PAFs was included, that based on a meta-analysis from Guh et al. (published by Zemp Stutz et al.) of 6.2 and 6.4%, respectively (10, 30).

Depending on the PAFs applied, the attributable fraction of the direct costs of asthma related to overweight and obesity in Switzerland 2021 are estimated at CHF 72–224 million overall, with CHF 35–116 million attributable to overweight and CHF 37–107 million attributable to obesity (see Table 3).

**Table 3:** Attributable fraction of overweight and obesity of the direct costs of seven selected obesity-related complications for Switzerland 2021 based on the annual changes of CPI in healthcare.

Disease	Direct cost of illness (Mio. CHF)^a^	PAF (%)		PAF-based cost (Million CHF)		
		Overweight 25 ≤ BMI < 30	Obesity BMI ≥ 30	Overweight 25 ≤ BMI < 30	Obesity BMI ≥ 30	Total Attributable direct costs BMI ≥ 25
Asthma	574 Based on Szucs et al. (40)	6.2	6.4	35	37	**72**
		20.3	18.7	116	107	**224**
CHD	1,861–2,022 Based on Destatis (41) and Wieser et al. (42)	6.6	4.7	123–133	87–95	**210–228**
		14.1	11.3	262–285	210–228	**473–513**
Depression	6,590 Based on Tomonaga et al. (43)	3.7	5.4	241	354	**595**
Diabetes mellitus	1,366 Based on Huber et al. (44)	16.2	42.9	221	586	**808**
		38.4	41.4	525	565	**1,090**
Hypertension	1,538 Based on Destatis (41)	18.1	16.8	278	258	**536**
Osteoarthritis (hip and knee)	2,830 Based on Destatis (45)	19.4^b^	19.6^b^	549	555	**1,104**
		24.4^c^	25.0^c^	690	707	**1,398**
Stroke	1,812–2,033 Based on Maercker et al. (46) and Luengo-Fernandez et al. (47)	5.9	5.4	108–121	98–110	**206–231**
		18.3	17.4	331–371	315–354	**646–725**
Overall				**1,555–2,506**	**1,954–2,594**	**3,530–5,081**

a Extrapolation of costs for Switzerland 2021 by conversion with PPP and extrapolation with CPI.

b Combined PAF based on relative risks for hip osteoarthritis from Holliday et al 2010 and knee osteoarthritis from Holliday et al. (32) and Muthuri et al. (33).

c Combined PAF based on relative risks for hip osteoarthritis and knee osteoarthritis both from Lohmander et al. (31).

BMI, Body mass index; CHD, Coronary heart disease; CPI, Consumer Price Index; PAF, Population attributable fraction. Bold values are overall values (sum).

#### 3.3.2 Coronary heart disease

CHD is the most common form of cardiovascular disease (CVD) and is responsible for 20% of deaths worldwide (48). For Switzerland, data on the number of patients hospitalized due to CVD events or the incidence of myocardial infarction (MI) are widely available. However, reliable data on the prevalence of CHD are scarce. Comparable data for Germany in 2017 estimated a total of 3.7% of women and 6.0% of men with CHD (defined as MI or chronic symptoms due to MI or angina) in the previous 12 months (49).

The costs of CHD are estimated using an indirect approach, as direct cost data are not available for Switzerland. The costs of acute and subsequent MI (ICD-10 I21 – I22) are used to extrapolate the costs of CHD (ICD-10 I20 – I25) based on the German cost structure, where MI accounts for 35.5% of the total costs of CHD in 2020 (41). In addition, it is assumed that the number of MI directly corresponds to the burden of CHD. Based on current Swiss population data, 18,966 cases of MI are to be expected in Switzerland 2021 (12,462 men and 6,506 women) (50). The costs of heart failure were not considered.

Taking into account the published cost data for acute and subsequent MI in Switzerland by Wieser et al., the extrapolated costs for MI in Switzerland in 2021 were estimated at CHF 718 million (including primary care, emergency and hospital care, rehabilitation and outpatient costs, see Supplementary Table S5) (42). Assuming that cost of MI is 35.5% of the total cost of CHD, the direct cost of CHD in Switzerland in 2021 is estimated to be CHF 2,022 million (41, 42). To account for uncertainty, the extrapolation and adjustment for Switzerland 2021 of the published German DESTATIS 2,020 data for Switzerland in 2021 yields a comparable figure of CHF 1,862 million (41).

PAFs for CHD due to overweight and obesity were calculated based on the risks reported by Flint et al. (US data 1986–2020) and the current overweight and obesity prevalence data from the SHS 2017, resulting in a PAF for overweight of 14.1% and a PAF for obesity of 11.3% (13, 26). In contrast to other studies, the reference category for BMI in the Flint et al. study was 18–22.9, which was adjusted accordingly in the calculation of the PAFs for this analysis. To consider the uncertainty, a second data source of PAFs was included based on the risks reported by Liu et al. (US 2012–2013), resulting in 6.6 and 4.7%, respectively, (see Table 1) (13, 27).

Depending on the PAFs applied, the attributable fraction of the direct costs of CHD related to overweight and obesity in Switzerland in 2021 is estimated to be CHF 210–513 million with CHF 123–285 million attributable to overweight and CHF 87–228 million attributable to obesity (see Table 3).

#### 3.3.3 Depression

In Switzerland in 2017, 5.3% of men, and 7.9% of women aged 15 years and older stated reported having suffered from depression in the past 12 months. The age group 55–64 years had the highest prevalence for both sexes (8.4% for men and 10.5% for women) (14, 51). Extrapolated to the permanent residential population of Switzerland in 2021, this corresponds to 493,364 inhabitants with depression (196,589 men; 296,775 women). Cost estimates and the distribution of severity grades of depression (21.5% mild, 46.9%moderate and 31.6% severe) in Switzerland were based on published data by Tomonaga et al. (43). Using the described extrapolation methods and population adjustment, the resulting direct costs of depression in Switzerland in 2021 were estimated at CHF 6,590 million over all severities (inpatient: CHF 5,040 million; outpatient: CHF 437 million; psychotherapy: CHF 739 million; prescribed medication CHF 373 million). The total costs for individual degrees of severity mild, moderate, and severe were CHF 486 million, 2,876 million and 3,228 million, respectively (see Supplementary Table S5).

PAFs for depression due to overweight and obesity were calculated based on the odds ratios reported by Luppino et al. (European and US data 2003–2008) and the current overweight and obesity prevalence data from the SHS 2017, resulting in a PAF for overweight of 3.7% and a PAF for obesity of 5.4% (see Table 1) (13, 28).

The attributable fraction of the direct costs of depression related to overweight and obesity in Switzerland in 2021 are estimated at CHF 595 million overall, with CHF 241 million attributable to overweight and CHF 354 million attributable to obesity (see Table 3).

#### 3.3.4 Diabetes mellitus

In 2017, 5.4% of men and 3.5% of women ≥15 years of age in Switzerland confirmed to have high blood sugar or to receive medication for diabetes (52). Extrapolated to the permanent residential population of Switzerland in 2021, this corresponds to 341,460 patients with diabetes (201,774 men; 139,685 women) (14, 52). The cost estimation of diabetes was based on data by Szucs et al. (reported in Schneider and Venetz 2012) (9). In addition to the extrapolation and population adjustment, the increase in prevalence of diabetes from 4.2% in 2012 to 4.4% in 2017 was considered as well (52).

PAFs for diabetes due to overweight and obesity were calculated based on the risks reported by Davin et al. from the Swiss CoLaus study (Swiss data 2003–2006), as BMI was calculated using standardized weight and height measurements, resulting in a PAF for overweight of 16.2% and a PAF for obesity of 42.9% (see Table 1) (29). A second data source of PAFs reported by Zemp Stutz et al. was included and amounted to 38.4 and 41.4%, respectively, (10).

The attributable fraction of the direct costs of diabetes related to overweight and obesity in Switzerland in 2021 are estimated at CHF 808–1,090 million overall, with CHF 221–525 million attributable to overweight and CHF 565–586 million attributable to obesity (see Table 3).

#### 3.3.5 Arterial hypertension

High blood pressure increases the risk of serious cardiovascular diseases, the most common cause of death in Switzerland in 2020. In 2017, 19.6% of men and 16.0% of women aged 15 years and older confirmed to have high blood pressure or to receive medication to lower their blood pressure with the highest prevalence in age group ≥75 years for both genders with over 55% of patients (53). Extrapolated to the permanent residential population of Switzerland in 2021, this would correspond to 1,371,955 inhabitants with high blood pressure (722,288 men; 649,667 women) (14, 53).

No publications reporting overall direct costs of hypertension based on Swiss data, which have been published after 2011, could be identified. As in available studies, the estimation of costs is limited to costs of drug therapy, using these data would underestimate the costs of the disease. For determining the full costs of hypertension, data from DESTATIS (Germany) was used (41). Considering the direct costs of disease from Germany in 2020, this will result in costs of disease for hypertension in Switzerland in 2021 of CHF 1,538 million (see Supplementary Table S5).

PAFs for hypertension due to overweight and obesity were calculated based on the risks reported by Davin et al. from the Swiss CoLaus study (Swiss data 2003–2006). BMI was calculated using standardized weight and height measurements, resulting in a PAF for overweight of 18.1% and a PAF for obesity of 16.8% (see Table 1) (29).

The attributable fraction of the direct costs of hypertension related to overweight and obesity in Switzerland in 2021 is estimated at CHF 536 million overall, with CHF 278 million attributable to overweight and CHF 258 million attributable to obesity (see Table 3).

#### 3.3.6 Osteoarthritis

Osteoarthritis is one of the most common outpatient diagnoses in general practice, internal medicine, or orthopedics and is defined by focal areas of articular cartilage loss within the synovial joints, associated with bone hypertrophy and joint capsule thickening, predominantly affecting hip and knee and to a lesser extent the hand, spine, foot or other joints (54).

No relevant publication could be identified, that estimated the cost of osteoarthritis in Switzerland. Therefore, costs are extrapolated based on German healthcare data. Both countries are similar, e.g., in the number of implantations and revisions of artificial knee joints (210 per 100,000 inhabitants in 2009) or hip joints, (290 per 100,000 inhabitants in 2009) (54). A German health survey in 2015 found that 17.9% of adults in Germany aged 18 years or older reported having osteoarthritis during the last 12 months, with higher prevalence among women (21.8%) than in men (13.9%) (55). The proportion of people with osteoarthritis increases with age; among people aged 65 years or older, almost half of the women (48.1%) and almost one third of men (31.2%) are affected (54, 55).

The direct costs for osteoarthritis (ICD-10 M15-M19), based on the German DESTATIS data, are estimated at CHF 2,830 million in Switzerland in 2021 (see Supplementary Table S5) (45, 54).

The PAFs for osteoarthritis of the hip or the knee due to overweight and obesity were calculated based on the risks reported by Lohmander et al. (31) (Sweden 1991–1996) and using the prevalence of overweight and obesity data from 2017 SHS. This results in PAFs of 13.3% for overweight and of 15.7% for obesity (see Table 1) (13, 31). To account for uncertainties in the epidemiologic data, a second source for PAF calculation was included and resulted in PAFs of 14.9 and 14.4% for hip osteoarthritis based on the risks reported by Holliday et al. (25) (UK 2002–2006) and PAFs of 23.8 and 24.7% for knee osteoarthritis based on the risks reported in a meta-analysis by Muthuri et al. including data from 1988 to 2010, respectively (13, 33).

The attributable fraction of the direct costs of osteoarthritis related to overweight and obesity in Switzerland in 2021 are thus estimated at CHF 1,104–1,398 million in total, with CHF 549–690 million attributable to overweight and CHF 555–707 million attributable to obesity depending on the PAFs applied (see Table 3).

#### 3.3.7 Stroke

In 2020, 21,041 people in Switzerland suffered a stroke (11,359 men; 9,682 women) (56). Extrapolated to the 2021 population, this equates to an incidence of stroke of 21,208 people in 2021 (11,453 for men and 9,755 for women). An Update of the EBC Study, that was considered in the analysis by Schneider and Venetz estimated, that inclusion of prevalent cases would result in 25% of the costs for stroke in addition to the incident cases (57). Extrapolating the Global Burden of Disease Study data, the prevalence of stroke in in Switzerland 2021 would result in 94,785 prevalent cases (58).

The cost estimate was based on total direct healthcare and non-medical care costs reported by Maercker et al. (Switzerland, Germany 2002–2004) (46). Extrapolation of these costs to the Swiss population with stroke (incident and prevalent) in the year 2021 resulted in direct costs of CHF 2033 million Due to the extrapolation of older data by Maercker et al. (46), a population-based cost analysis of European countries by Luengo-Fernandez et al. (Switzerland 2017) was included to consider the uncertainty evaluating the costs of stroke (including costs for overall healthcare, social care, and informal care), which amounted to CHF 1,812 million after extrapolation for Switzerland 2021 (see Supplementary Table S5) (47).

PAFs for stroke due to overweight and obesity were calculated based on the RR reported by Winter et al. (Germany 2005–2006) using the overweight and obesity prevalence from the SHS from 2017. PAFs resulted in 18.3% for overweight and 17.4% for obesity (see Table 1) (13, 34). To consider the uncertainty, an additional PAF published by Zemp Stutz et al. was included and amounted to 5.9 and 5.4%, respectively (10).

The attributable fraction of the direct costs of stroke related to overweight and obesity in Switzerland in 2021 is estimated at CHF 206–725 million In total, with CHF 108–371 million attributable to overweight and CHF 98–354 million attributable to obesity (see Table 3).

## 4 Discussion

**Figure 2:**
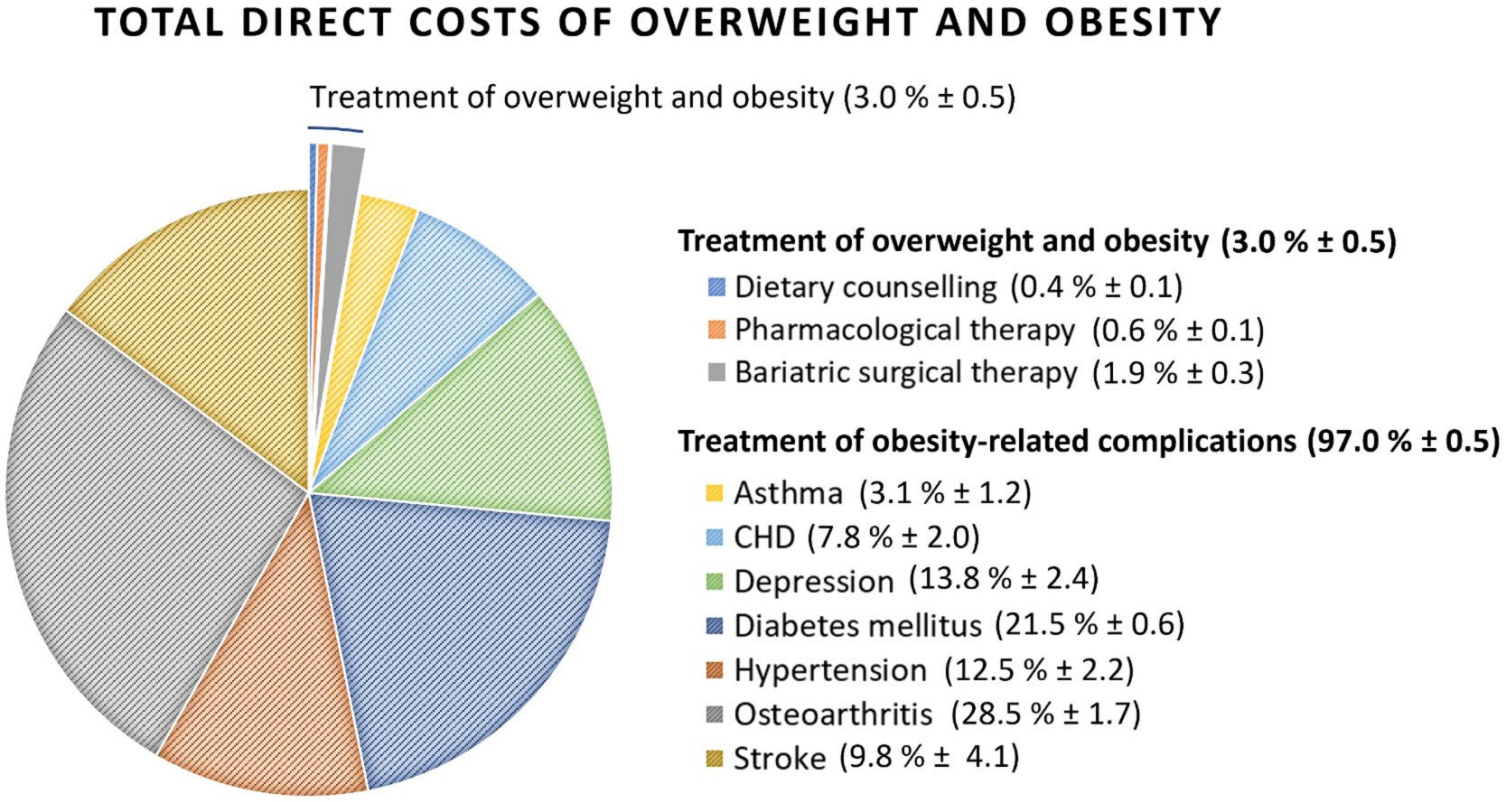
Total direct costs of treatment of overweight and obesity and related complications in Switzerland 2021.

The estimated total cost for overweight and obesity in Switzerland is CHF 3,656–5,207 million. The vast majority of these costs (97–98%) were accounted for by the seven obesity-related complications (CHF 3,530–5,081, see Figure 2). Direct treatment costs for overweight and obesity were 1.5–2.3% for bariatric surgery (CHF 83 million), 0.3–0.7% for the most recent pharmacological therapies (CHF 26 million) and 0.2–0.5% for dietary counseling (CHF 18 million), respectively (Table 2). While accounting for only a small proportion of total costs, pharmacological therapies, especially those with GLP-1 receptor agonists, represent a highly effective tool in management of overweight and obesity (59). Noteworthy, based on market analyses, a significant portion of the cost of pharmacological therapy is not covered by the OKP and is instead borne by the patients themselves, partly due to legal regulations on cost coverage, experienced feelings of shame, or simplified access in combination with an individually perceived high medical need.

**Table 2:** Total costs of overweight and obesity for Switzerland 2021 based on the annual changes of CPI in healthcare.

Type of costs	Costs (Million CHF)
Direct costs of treatment overweight and obesity	
Dietary counseling costs^a^	18
Pharmacological therapy costs^b^	26
Bariatric surgical therapy costs^c^	83
**Total direct costs of treatment overweight and obesity**	**126**
Direct costs of obesity-linked selected overweight and obesity-related complications	
Asthma	72–224
CHD	210–513
Depression	595
Diabetes mellitus	808–1,090
Hypertension	536
Osteoarthritis (hip & knee)	1,104–1,398
Stroke	206–725
**Total direct costs attributable to overweight and obesity**	**3,530–5,081**
**Total costs of overweight and obesity**	**3,657–5,208**

a Costs for dietary counseling were estimated by the most current statistical information of Jan-Jun 2022.

b Costs for pharmacological therapy were estimated by the most current sales information of Jan-Jun 2022.

c Costs for bariatric surgical therapy costs were estimated based on the number of procedures performed in 2019, as the COVID-19 pandemic led to a significant reduction in elective surgical procedures performed in 2020 and 2021.

CHD, Coronary heart disease; CPI, Consumer Price Index; PAF, Population attributable fraction. Bold values are overall values (sum).

The direct costs of overweight and obesity in Switzerland in 2021, estimated in this analysis, range from 0.49 to 1.07% of Swiss gross domestic product (GDP) in 2021, depending on the method used to account for inflation. Okunogbe et al. estimated the total burden of overweight and obesity, including direct and indirect costs for Switzerland at 2.0% of its GDP in 2019 (assuming that 50.3% of the population has overweight or obesity) (60). These costs include direct medical and non-medical expenditures for the treatment of obesity and related diseases, with direct costs accounting for only 38.2% of the total costs, while 61.8% is due to absenteeism, presenteeism, and premature mortality.

Considering Okunogbe et al.’s estimates, this implies that the direct costs of overweight and obesity are approximately 0.76% of Swiss GDP in 2019. This is consistent with the reported direct costs estimated in this study for 2021. Okunogbe et al. further estimated the average total health economic burden of obesity in high-income countries to be 2.46% of GDP in 2019, rising to 2.88% in 2030 and 3.8% in 2060 (60).

On average, 53.1% of Europeans are considered to have overweight or obesity compared to 41.9% of Swiss inhabitants. Although Switzerland reports a lower obesity prevalence of 11.3% compared to the European average of 16.8%, there is reason for concern. A unidirectional trend toward an increase of both overweight and obesity is reported for each country included in the aforementioned OECD analysis, including Switzerland, which should be addressed with the necessary vigor.

These findings underscore the significant challenges that overweight and obesity present for individual and public health. At the patient level, there are direct consequences, including stigmatization and a negative impact on various aspects of quality of life. Additionally, overweight and obesity significantly increase the risk of developing a variety of non-communicable diseases, which has a significant impact on personal health, as well as on the consumption of healthcare resources and thus on the costs to the healthcare system and society. The results of the present study are important for two reasons. First, they provide the most recent information available on the costs of overweight and obesity in Switzerland. Second, they indicate that the cause of this substantial contribution to healthcare costs and OKP expenditures can potentially be influenced, by measures targeting the reduction of overweight and obesity.

However, the monetary magnitude of this identified preventive potential is subject to the scope and methods applied. Firstly, only the costs of seven selected obesity-related complications were considered, despite the fact that there are in excess of 50 diseases associated with overweight and obesity (e.g., anxiety, sleep apnea, non-alcoholic liver disease and non-alcoholic steatohepatitis, gallstones, gout, infertility, incontinence, chronic back pain as well as malignant neoplasms of breast, colon, kidney or other cancers) (61). Second, it should be noted that some relevant healthcare costs were not considered in this analysis. These include alternative therapeutic interventions, diagnostic procedures, consultations with healthcare professionals, accompanying therapies as well as follow-up examinations and long-term complications such as micronutrient supplementation after bariatric surgery and more. In addition, the estimate does not include cost of psychological therapy for overweight and obesity-related mental health problems other than depression, which have a high prevalence in this population (62). Third, the focus of this study was on the direct costs associated with the management of overweight and obesity as well as seven related complications. Indirect costs, which typically include productivity losses as well as absenteeism, have not been included, as they typically play a lesser role in public decision-making due to their inherent challenges in accurately quantifying and weighing such costs. However, estimates by Okunogbe et al. suggest that the indirect costs of overweight and obesity are approximately 1.6 times the direct costs (60). Fourth, the cost estimate is based on self-reported BMI of the 2017 SHS data, which is prone to underestimate body weight and overestimate body height in individuals with overweight or obesity (29, 63, 64). To address this uncertainty, an additional analysis was performed with BMI data from menuCH in 2014, based on standardized weight and height measurements, which indicated an even higher prevalence of both overweight and obesity (65, 66). Overall, the health economic burden presented in our study represents is a *de facto* conservative estimate of the cost of overweight and obesity in Switzerland.

Beyond the cost estimate, there is a second key finding: the available health economic evidence is scarce. Most of the sources used in the 2012 report are still the most relevant today. This is particularly disappointing as respective primary data are available but to this day are unpublished or hardly accessible. Given the scarcity of evidence, a standard procedure for updating and estimating costs was not feasible due to the substantial variation in disease-specific evidence. Substantial conversions, extrapolations, and adjustments were therefore necessary to estimate the direct costs of overweight and obesity and related comorbidities for Switzerland in 2021. In particular, the reported PAFs vary widely, leading to large variations and uncertainty in the costs of overweight and obesity-related complications in Switzerland.

Another assumption relates to the extrapolation of costs from the respective source year of the available data to the year 2021. To extrapolate costs from a source year to 2021, annual changes in total health expenditures have been used previously (9). However, this method is considered to be a rough approximation as it includes expansion of services, which may overcompensate for price changes while not considering improvements in treatment pattern, e.g., due to changes in treatment paradigms or newly approved pharmaceuticals and technologies. Therefore, for this analysis, the annual changes in the relevant subcodes of the consumer price index in healthcare were used instead to better incorporate changes of individual cost components (23). Changes in this index account only for changes in prices and tariffs, but not volume changes. The annual change of health expenditures was used for information purposes only, to allow for comparison with previous estimates and are presented in Supplementary Tables S6, S7.

Despite the heterogeneous data and the scarcity of evidence, two main conclusions can be drawn from this study. First, regardless of the exact figures and considering only the results of this conservative approach, the estimated direct costs of overweight and obesity and related complications have a substantial impact on the Swiss healthcare system, accounting for 4.4–6.3% of total healthcare expenditures. Although direct management of overweight and obesity with dietary counseling, pharmacologic therapy, and bariatric surgery accounts for only 0.15% of the total Swiss healthcare expenditures, reduction of the prevalence of overweight and obesity would significantly lower the costs of treating obesity-related complications and thus reduce the burden and costs of overweight and obesity in Switzerland. This is of considerable significance, as the cause for these expenditures can be reduced through treatment and prevention. A recently published systematic review and meta-analysis demonstrated that therapeutic patient education interventions result in significant improvements in several health indicators among patients with obesity and the efficacy of these interventions was confirmed across a range of biomedical, psychosocial and psychological outcomes (67).

While other countries have implemented stricter regulations such as sugar taxes, Switzerland’s approach emphasizes voluntarism. In addition, preventive measures regarding overweight and obesity are scarce in Switzerland and vary widely in their allocation and implementation, as they are the responsibility of the individual cantons. While some preventive measures for children and adolescents are successfully implemented at a local level, there is a notable lack of provision for adults. It is therefore essential to facilitate the adoption and implementation of preventive measures at the national level for Switzerland in the future.

Secondly, there is a paucity of health economic evidence, which is largely outdated, both in Switzerland and internationally. The opportunities offered by the digitization for health economic and health services research have not yet been fully exploited. Inadequate use of available data for research, information, and decision making creates high uncertainty for prioritization and impact assessment in health policy. Missing or incomplete disclosure of data ultimately leads to a lack of transparency, which in turn leads to a significant information asymmetry, as access to this information is primarily restricted to health insurers.

Recommendations and proposals for the introduction of multidisciplinary programs for the treatment of people with obesity have been formulated for Switzerland (68). However, in the face of ever-increasing rates of overweight and obesity, a major effort is needed in scientific research to develop effective prevention and treatment strategies. The urgency of this matter cannot be overstated. Investing in both scientific research and research-guided adaptation of preventive measures should be accorded a high priority in order to combat obesity.

## Data availability statement

The original contributions presented in the study are included in the article/Supplementary material, further inquiries can be directed to the corresponding author.

## Author contributions

DS: Conceptualization, Investigation, Methodology, Writing – original draft, Writing – review & editing. PH: Conceptualization, Investigation, Methodology, Project administration, Writing – original draft, Writing – review & editing. SR: Conceptualization, Investigation, Methodology, Writing – original draft, Writing – review & editing. DF: Validation, Writing – review & editing. ZP: Validation, Writing – review & editing. RP: Validation, Writing – review & editing. BS: Validation, Writing – review & editing. SL: Funding acquisition, Methodology, Writing – review & editing. TP: Funding acquisition, Methodology, Writing – review & editing.
