# Supplementary material for: Cost of overweight, obesity, and related complications in Switzerland 2021
Source: Front Public Health. 2024 Jul 12;12:1335115. doi: 10.3389/fpubh.2024.1335115 (PMC11282501; doi:10.3389/fpubh.2024.1335115)
Supplement: Supplementary file 1 [file Data_Sheet_1.docx]

Supplementary Material

# Supplementary Figures and Tables

Supplementary Table 1: Number of extrapolated overall dietary consultations in Switzerland in 2022 [21]

| Tariff code | Average monthly consultations  in January-June 2022 | Extrapolated consultations  for 2022 | Estimated consultations for 2022 attributable to overweight/obesity^a^ | Cost per consulation^a^ | Costs for 2022  [CHF]^b^ |  |
| --- | --- | --- | --- | --- | --- | --- |
| Outpatient dietary consultations (total) | | **241 086** | **192 869** | **-** | **15 370 411** |  |
| 7811 – First consultation | 4 870 | 58 434 | 46 747 | 99 | 2 892 483 |  |
| 7812 – 2nd to 6th consultation | 11 369 | 136 428 | 109 142 | 77 | 5 252 478 |  |
| 7813 – 7th consultation or more | 3 763 | 45 156 | 36 125 | 64 | 1 444 992 |  |
| 7814 – Group meetings | 89 | 1 068 | 854 | 31 | 16 554 |  |
| Inpatient dietary consultations (total) | | **188 062** | **37 612** | **-** | **2 953 397** |  |
| 7821 – First consultation | 4 260 | 51 124 | 10 225 | 99 | 2 530 638 |  |
| 7822 – 2nd to 6th consultation | 8 095 | 97 136 | 19 427 | 77 | 3 739 736 |  |
| 7823 – 7th consultation or more | 2 506 | 30 072 | 6 014 | 64 | 962 304 |  |
| 7824 – Group meetings | 811 | 9 730 | 1 946 | 31 | 150 815 |  |
| Total dietary consultations (total) | | **429 148** | **230 481** | **-** | **18 323 808** |  |
| a: Assuming a proportion of overweight and obesity related consultations of approx. 80 % for outpatient dietary consultations and approx. 20 % of inpatient dietary consultations.  b: Cost per consultation is based on tax points [22, 23]. As of Nov 2022, one tax point equals one Swiss Franc [24, 25]. | | | | | | |

Supplementary Table 2: CHOP Codes, description and number of bariatric procedures carried out in 2019 [21]

| CHOP-code | Description | Total procedures 2019 |
| --- | --- | --- |
| Gastric bypass (total) | | **3 630** |
| 44.31.09 | Gastric bypass (others) | 0 |
| 44.31.11-16 | Gastric bypass proximal | 2 046 |
| 44.31.21-22 | Gastric bypass distal | 82 |
| 44.31.31-32 | Gastric bypass Omega-loop | 29 |
| 44.31.41-42 | Gastric bypass Roux-en-Y | 871 |
| 44.5X.21-36  44.5X.51-52 | Gastric bypass Revision | 290 |
| 45.97.11-12 | Dista-/proximalisation after gastric bypass | 122 |
| 43.99.10-11 | Gastrectomy after gastric bypass | 3 |
| 46.99.10-11 | Re-intervention for internal hernia (or suspicion of) | 187 |
| Sleeve resection (total) | | **1 007** |
| 43.89.40-41 | Sleeve resection (not complex) | 964 |
| 43.89.42-45 | Sleeve resection (complex; after banding or resleeve) | 43 |
| Restrictive gastric procedure (total) | | **273** |
| 44.95.11-12 | Gastric Banding | 95 |
| 44.96.11-14 | Gastric Banding repositioning/change | 22 |
| 44.97.11-12 | Gastric Banding removal | 145 |
| 44.95.21-22 | Vertical banded gastroplasty | 1 |
| 44.96.99 | Vertical banded gastroplasty restapling | 10 |
| Biliopancreatic diversion (total) | | **13** |
| 43.89.20-21 | Biliopancreatic diversion (Scopianaro) | 0 |
| 43.89.30-31 | Biliopancreatic diversion with duodenal switch | 0 |
| 43.89.50-51 | Biliopancreatic diversion with duodenal switch after sleve resection | 2 |
| 44.5X.41-44 | Biliopancreatic diversion revision/reversal | 11 |
| Other procedures | | **3** |
| 44.68 | Laparoscopic gastroplasy | 3 |
| Overall procedures | | **4 926** |

Supplementary Table 3**:** Number of performed bariatric surgeries 2019 depending on the billed DRG [21]

| Group | Type of intervention | CHOP19 Code | Bariatric procedure | Complex bariatric procedure | SMOB Code | MDC 6 | | | | | | | | | | | MDC 7 | | MDC 21B | Total number procedures 2019 | Average cost for procedure |
| --- | --- | --- | --- | --- | --- | --- | --- | --- | --- | --- | --- | --- | --- | --- | --- | --- | --- | --- | --- | --- | --- |
|  |  |  |  |  |  | DRG [G12A](https://datenspiegel110.swissdrg.org/drgs/6152f58a1cc62462bad663b1?locale=de) | DRG [G12B](https://datenspiegel110.swissdrg.org/drgs/6152f58a1cc62462bad663b2?locale=de) | DRG [G18A](https://datenspiegel110.swissdrg.org/drgs/6152f58a1cc62462bad6637b?locale=de) | DRG [G18B](https://datenspiegel110.swissdrg.org/drgs/6152f58a1cc62462bad6637c?locale=de) | DRG [G19A](https://datenspiegel110.swissdrg.org/drgs/6152f58a1cc62462bad6637d?locale=de) | DRG [G19B](https://datenspiegel110.swissdrg.org/drgs/6152f58a1cc62462bad6637e?locale=de) | DRG [G19C](https://datenspiegel110.swissdrg.org/drgs/6152f58a1cc62462bad6637f?locale=de) | DRG [G33Z](https://datenspiegel110.swissdrg.org/drgs/6152f58a1cc62462bad6638c?locale=de) | DRG [G38A](https://datenspiegel110.swissdrg.org/drgs/6152f58a1cc62462bad6638f?locale=de) | DRG [G38B](https://datenspiegel110.swissdrg.org/drgs/6152f58a1cc62462bad66390?locale=de) | DRG [H12A](https://datenspiegel110.swissdrg.org/drgs/6152f58a1cc62462bad663e5?locale=de) | | DRG H33Z | DRG [X06B](https://datenspiegel110.swissdrg.org/drgs/6152f58d1cc62462bad667a4?locale=de) |  |  |
| Gastric banding (total) | | | | | | **2** | **76** | **1** | **46** | **3** | **120** | **6** | **1** | **0** | **1** | **0** | | **0** | **6** | **262** | 15.440,58 CHF |
| Gastric band insertion | Gastric Banding open | 44.95.11 | x |  | 1001 |  |  |  |  |  |  |  |  |  |  |  | |  |  | 0 |  |
|  | Gastric Banding laparoscopic | 44.95.12 | x |  | 1002 | 1 | 11 |  | 32 | 1 | 47 | 2 | 1 |  |  |  | |  |  | 95 |  |
| Gastric band removal | Gastric band Removal open | 44.97.11 |  |  | 1011 |  |  |  |  |  | 3 |  |  |  |  |  | |  |  | 3 |  |
|  | Gastric band Removal laparoscopic | 44.97.12 |  |  | 1012 |  | 54 |  | 8 | 2 | 69 | 4 |  |  |  |  | |  | 5 | 142 |  |
| Gastric band reposition | Band repositioning after Gastric Banding open | 44.96.11 | x | x | 1021 |  |  |  |  |  |  |  |  |  |  |  | |  |  | 0 |  |
|  | Band repositioning after Gastric Banding laparoscopic | 44.96.12 | x | x | 1022 | 1 | 3 | 1 | 2 |  |  |  |  |  |  |  | |  | 1 | 8 |  |
| Gastric band change | Band Change after Gastric Banding open | 44.96.13 | x | x | 1031 |  |  |  |  |  |  |  |  |  |  |  | |  |  | 0 |  |
|  | Band Change after Gastric Banding laparoscopic | 44.96.14 | x | x | 1032 |  | 8 |  | 4 |  | 1 |  |  |  | 1 |  | |  |  | 14 |  |
| Gastric bypass (total) | | | | | | **0** | **0** | **50** | **376** | **19** | **2.936** | **0** | **30** | **27** | **1** | **4** | | **0** | **0** | **3.630** | 17.130,18 CHF |
| Gastric Bypass | Proximal gastric bypass after open fundoplication | 44.31.09 | x | x | 2041 |  |  |  |  |  |  |  |  |  |  |  | |  |  | 0 |  |
|  | Proximal gastric bypass after fundoplication laparoscopically | 44.31.09 | x | x | 2042 |  |  |  |  |  |  |  |  |  |  |  | |  |  | 0 |  |
|  | Proximal gastric bypass (alimentary limb <150cm) open | 44.31.11 | x |  | 2001 |  |  |  | 1 |  | 7 |  |  |  |  |  | |  |  | 8 |  |
|  | Proximal Gastric Bypass (alimentary limb <150cm) laparoscopic | 44.31.12 | x |  | 2002 |  |  | 3 | 26 | 5 | 1.762 |  | 6 | 6 |  |  | |  |  | 1808 |  |
|  | Proximal gastric bypass after sleeve gastrectomy open | 44.31.13 | x | x | 2011 |  |  |  |  | 1 | 7 |  |  |  |  |  | |  |  | 8 |  |
|  | Proximal gastric bypass after sleeve gastrectomy laparoscopic | 44.31.14 | x | x | 2012 |  |  | 2 | 1 | 2 | 145 |  | 4 |  |  |  | |  |  | 154 |  |
|  | Proximal gastric bypass after open gastric banding | 44.31.15 | x | x | 2021 |  |  |  |  |  | 4 |  |  |  |  |  | |  |  | 4 |  |
|  | Proximal gastric bypass after vertical banded gastroplasty open | 44.31.15 | x | x | 2031 |  |  |  |  |  |  |  |  |  |  |  | |  |  | 0 |  |
|  | Proximal gastric bypass after gastric banding laparoscopic | 44.31.16 | x | x | 2022 |  |  |  | 1 | 1 | 61 |  |  |  | 1 |  | |  |  | 64 |  |
|  | Proximal gastric bypass after vertical banded gastroplasty laparoscopic | 44.31.16 | x | x | 2032 |  |  |  |  |  |  |  |  |  |  |  | |  |  | 0 |  |
|  | Distal gastric bypass (common channel ≤ 100 cm) open | 44.31.21 | x | x | 2051 |  |  |  |  |  | 2 |  |  |  |  |  | |  |  | 2 |  |
|  | Distal gastric bypass (common channel ≤ 100 cm) laparoscopic | 44.31.22 | x | x | 2052 |  |  | 2 | 3 | 1 | 74 |  |  |  |  |  | |  |  | 80 |  |
|  | Roux-en-Y gastric bypass (alimentary limb <150cm) open | 44.31.41 | x |  | 2001 |  |  |  | 2 | 3 | 6 |  | 1 |  |  |  | |  |  | 12 |  |
|  | Roux-en-Y gastric bypass (alimentary limb <150cm) laparoscopic | 44.31.42 | x |  | 2002 |  |  | 3 | 5 | 4 | 842 |  | 2 | 2 |  | 1 | |  |  | 859 |  |
| Gastric Bypass Revisions | Reversal after open gastric bypass | 44.5X.51 | x | x | 2061 |  |  |  | 1 |  |  |  |  |  |  |  | |  |  | 1 |  |
|  | Reversal after gastric bypass laparoscopic | 44.5X.52 | x | x | 2062 |  |  |  | 4 |  |  |  | 1 | 1 |  |  | |  |  | 6 |  |
|  | Pouch revision after gastric bypass without new pouch open | 44.5X.33 | x | x | 2071 |  |  |  | 2 |  |  |  |  | 2 |  |  | |  |  | 4 |  |
|  | Pouch revision after gastric bypass without new laparoscopic device | 44.5X.34 | x | x | 2072 |  |  | 5 | 72 |  |  |  |  |  |  |  | |  |  | 77 |  |
|  | Pouch revision after gastric bypass with new appliance open (incl. Fobi ring) | 44.5X.35 | x | x | 2073 |  |  |  | 4 |  |  |  | 1 | 2 |  |  | |  |  | 7 |  |
|  | Pouch revision after gastric bypass with new laparoscopic installation (incl. fobi ring) | 44.5X.36 | x | x | 2074 |  |  | 6 | 66 |  |  |  |  | 1 |  |  | |  |  | 73 |  |
|  | Anastomosis revision after gastric bypass open (incl. correction of loop length) | 44.5X.21 | x | x | 2081 |  |  | 6 | 13 |  |  |  | 2 | 3 |  |  | |  |  | 24 |  |
|  | Anastomosis revision after gastric bypass laparoscopic (incl. correction of loop length) | 44.5X.22 | x | x | 2082 |  |  | 14 | 82 |  |  |  |  | 2 |  |  | |  |  | 98 |  |
| Gastrektomy after Gastric Bypass | Residual gastrectomy after open gastric bypass | 43.99.10 | x | x | 2091 |  |  | 2 |  |  |  |  | 1 |  |  |  | |  |  | 3 |  |
|  | Residual gastrectomy after gastric bypass laparoscopic | 43.99.11 | x | x | 2092 |  |  |  |  |  |  |  |  |  |  |  | |  |  | 0 |  |
| Reintervention for inner hernia | Reintervention for inner hernia (or suspicion) open | 46.99.93 |  |  | 2101 |  |  | 18 | 28 |  |  |  | 1 | 5 |  |  | |  |  | 52 |  |
|  | Reintervention for inner hernia (or suspicion) laparoscopic | 46.99.94 |  |  | 2102 |  |  | 7 | 88 | 1 | 34 | 3 | 1 | 1 |  |  | |  |  | 135 |  |
| Distalisation after Gastric Bypass | Distalisation after proximal gastric bypass open | 45.97.11 | x | x | 2111 |  |  | 5 | 19 |  |  |  | 7 | 8 |  | 2 | |  |  | 41 |  |
|  | Distalisation after proximal gastric bypass laparoscopic | 45.97.12 | x | x | 2112 |  |  | 2 | 74 | 1 |  |  | 4 |  |  |  | |  |  | 81 |  |
|  | Proximalisation after distal gastric bypass open | 45.97.11 | x | x | 2121 |  |  |  |  |  |  |  |  |  |  |  | |  |  | 0 |  |
|  | Proximalisation after distal gastric bypass laparoscopically | 45.97.12 | x | x | 2122 |  |  |  |  |  |  |  |  |  |  |  | |  |  | 0 |  |
| Gastric Mini-Bypass | Omega-loop gastric bypass (mini gastric bypass) open | 44.31.31 | x | x | 2501 |  |  |  |  |  | 2 |  | 1 |  |  | 1 | |  |  | 4 |  |
|  | Omega-loop gastric bypass (mini gastric bypass) laparoscopic | 44.31.32 | x | x | 2502 |  |  |  |  | 1 | 24 |  |  |  |  |  | |  |  | 25 |  |
| Sleeve Gastrectomy (total) | | | | | | **0** | **0** | **0** | **4** | **19** | **963** | **0** | **3** | **12** | **6** | **0** | |  | **0** | **1.007** | 16.641,20 CHF |
| Sleeve Gastrektomie | Sleeve gastrectomy open | 43.89.40 | x |  | 3001 |  |  |  |  | 2 | 3 |  | 1 | 6 | 1 |  | |  |  | 13 |  |
|  | Sleeve gastrectomy laparoscopic | 43.89.41 | x |  | 3002 |  |  |  | 3 | 17 | 919 |  | 2 | 5 | 5 |  | |  |  | 951 |  |
|  | Sleeve gastrectomy after Gastric band open | 43.89.42 | x | x | 3011 |  |  |  |  |  | 2 |  |  |  |  |  | |  |  | 2 |  |
|  | Sleeve gastrectomy after Gastric band laparoscopic | 43.89.43 | x | x | 3012 |  |  |  |  |  | 18 |  |  |  |  |  | |  |  | 18 |  |
|  | Re-sleeve gastrectomy open | 43.89.44 | x | x | 3021 |  |  |  |  |  |  |  |  |  |  |  | |  |  | 0 |  |
|  | Re-sleeve gastrectomy laparoscopic | 43.89.45 | x | x | 3022 |  |  |  | 1 |  | 21 |  |  | 1 |  |  | |  |  | 23 |  |
| Biliopancreatic diversion (total) | | | | | | **0** | **0** | **3** | **7** | **0** | **2** | **0** | **0** | **1** | **0** | **0** | |  | **0** | **13** | 25.043,50 CHF |
| Biliopancreatic diversion | Scopinaro Typ BPD open | 43.89.20 | x | x | 4001 |  |  |  |  |  |  |  |  |  |  |  | |  |  | 0 |  |
|  | Scopinaro Typ BPD laparoscopic | 43.89.21 | x | x | 4002 |  |  |  |  |  |  |  |  |  |  |  | |  |  | 0 |  |
|  | BPD-duodenal switch open | 43.89.30 | x | x | 4011 |  |  |  |  |  |  |  |  |  |  |  | |  |  | 0 |  |
|  | BPD-duodenal switch laparoscopic | 43.89.31 | x | x | 4012 |  |  |  |  |  |  |  |  |  |  |  | |  |  | 0 |  |
|  | BPD-duodenal switch after Sleeve gastrectomy open | 43.89.50 | x | x | 4021 |  |  |  |  |  |  |  |  |  |  |  | |  |  | 0 |  |
|  | BPD-duodenal switch after Sleeve gastrectomy laparoscopic | 43.89.51 | x | x | 4022 |  |  |  |  |  | 2 |  |  |  |  |  | |  |  | 2 |  |
| Biliopancreatic diversion Revision/reversal | Reversal after BPD open | 44.5X.43 | x | x | 4031 |  |  | 1 |  |  |  |  |  |  |  |  | |  |  | 1 |  |
|  | Reversal after BPD laparoscopic | 44.5X.44 | x | x | 4032 |  |  |  |  |  |  |  |  |  |  |  | |  |  | 0 |  |
|  | Revision after BPD (incl. extension common channel, sleeve size) open | 44.5X.41 | x | x | 4041 |  |  |  | 3 |  |  |  |  | 1 |  |  | |  |  | 4 |  |
|  | Revision after BPD (incl. extension of common channel, sleeve size) laparoscopic | 44.5X.42 | x | x | 4042 |  |  | 2 | 4 |  |  |  |  |  |  |  | |  |  | 6 |  |
| Vertical banded gastroplasty (total) | | | | | | **0** | **6** | **0** | **2** | **0** | **1** | **1** | **0** | **1** | **0** | **0** | |  | **0** | **11** | 18.707,23 CHF |
| Vertical banded gastroplasty | Vertical gastroplasty (vertical ligament gastroplasty) open | 44.95.21 | x |  | 5001 |  |  |  |  |  |  |  |  |  |  |  | |  |  | 0 |  |
|  | Vertical gastroplasty (vertical band gastroplasty) laparoscopic | 44.95.22 | x |  | 5002 |  |  |  |  |  |  | 1 |  |  |  |  | |  |  | 1 |  |
| Vertical banded gastroplasty Restapling | Reattachment according to VBG open | 44.96.99 | x | x | 5011 |  |  |  | 2 |  | 1 |  |  | 1 |  |  | |  |  | 4 |  |
|  | Reattachment after VBG laparoscopic | 44.96.99 | x | x | 5012 |  | 6 |  |  |  |  |  |  |  |  |  | |  |  | 6 |  |
| Other procedures (total) | | | | | |  |  |  |  |  | 1 | 1 | 1 |  |  |  | |  |  | **3** | 28.117,30 CHF |
| Laparoscopic gastroplasty | Laparoscopic gastroplasty | 44.68 |  |  | - |  |  |  |  |  | 1 | 1 | 1 |  |  |  | |  |  | 3 |  |
| Number Procedures in DRG | | | | | | **2** | **82** | **79** | **551** | **42** | **4.057** | **11** | **37** | **47** | **8** | **4** | | **0** | **6** | **4.926** |  |

Supplementary Table 4: Cost estimate of bariatric surgical procedures carried out in Switzerland 2019 [21]

| DRG | Number of procedures | Median cost of procedure in DRG  [2019 CHF] | Total Cost for procedures in DRG  [2019 CHF] |
| --- | --- | --- | --- |
| G12A | 2 | 37 811.40 | 75 623 |
| G12B | 82 | 11 912.05 | 976 788 |
| G18A | 79 | 31 761.00 | 2 509 119 |
| G18B | 551 | 18 868.40 | 10 396 488 |
| G19A | 42 | 30 578.55 | 1 284 299 |
| G19B | 4 057 | 15 373.90 | 62 371 912 |
| G19C | 11 | 13 740.60 | 151 147 |
| G33Z | 37 | 55 237.40 | 2 043 784 |
| G38A | 47 | 67 455.95 | 3 170 430 |
| G38B | 8 | 53 495.60 | 427 965 |
| H12A | 4 | 39 206.80 | 156 827 |
| X06B | 6 | 6 167.50 | 37 005 |
| Total | **4 926** | - | **83 601 387** |

Supplementary Table 5: Direct costs of selected overweight and obesity related complications and their adaptation to Swiss conditions for 2021. Extrapolation of costs for Switzerland 2021 by conversion with PPP and extrapolation with CPI

| Type of cost | Reference | Country | Cost year | Source cost  [in Million] | Extrapolation for 2021 [Million CHF] | Population-adjusted cost for 2021 [Million CHF] |
| --- | --- | --- | --- | --- | --- | --- |
| Asthma | | | | | | |
| Overall direct cost of asthma | Szucs et al 1999^a^ [13]. | CH | 1997 | **CHF 1 778.00 per patient** | **CHF 1 520.07 per patient** | **574** |
| Inpatient cost |  |  |  | CHF 826.00 per patient | CHF 902.96 per patient | 341 |
| Outpatient cost |  |  |  | CHF 210,00 per patient | CHF 205.04 per patient | 77 |
| Prescribed medication |  |  |  | CHF 731,00 per patient | CHF 412.07 per patient | 156 |
| CHD | | | | | | |
| Overall direct cost of MI^b^ (ICD‑10 I21-I22) | Wieser et al 2012 [14] | CH | 2008 | **CHF 514.** | **484** | **718^c^** |
| Primary care |  |  |  | CHF 8. | 8 | 11^c^ |
| Emergency and hospital care |  |  |  | CHF 236. | 222 | 330^c^ |
| Rehabilitation |  |  |  | CHF 23. | 22 | 32^c^ |
| Outpatient (incl. pharmaceuticals) |  |  |  | CHF 247. | 232 | 345^c^ |
| Overall direct cost of CHD (ICD‑10 I20-I25) | DESTATIS 2020 [15] | DE | 2020 | **EUR 7 946** | **17 713** | **1 862** |
| Overall direct cost of MI (ICD‑10 I21-I22) | DESTATIS 2020 [15] | DE | 2020 | **EUR 2 823** | **6 293** | **661** |
| Overall direct cost of acute MI (ICD‑10 I21) |  |  |  | EUR 2 814 | 6 273 | 659 |
| Overall direct cost of subsequent MI (ICD‑10 I22) |  |  |  | EUR 9 | 20 | 2 |
| Diabetes mellitus | | | | | | |
| Overall direct cost of diabetes mellitus | Szucs et al. 2000 [12]. | CH | 2011 (extrapol. from 2000) | **CHF 1 315** | **1 241** | **1.366** |
| Depression | | | | | | |
| Overall direct cost of depression (all grades) | Huber et al. 2014^a^ [17] | CH | 2007-2008 | **EUR 3 800** | **6 835** | **6.590** |
| Mild depression |  |  |  | EUR 3 561.00 per patient | CHF 4 580.30  per patient | 486 |
| Moderate depression |  |  |  | EUR 9 744.00 per patient | CHF 12 429.41 per patient | 2 876 |
| Severe depression |  |  |  | EUR 16 239.00  per patient | CHF 20 706.03 per patient | 3 228 |
| Hypertension | | | | | | |
| Overall direct cost of hypertension (ICD-10 I10-I15 | DESTATIS 2022 [15] | DE | 2020 | **EUR 6 566.** | **14 696** | **1 538** |
| Osteoarthritis | | | | | | |
| Overall direct cost of osteoarthritis (ICD‑10 M15-M19) | DESTATIS 2022 [18] | DE | 2020 | **EUR 12 079** | **26 927** | **2 830** |
| Stroke | | | | | | |
| Total direct cost of stroke (ICD-10 code I61, I63, I64, I67)^d^ | Maerker et al. 2013^a^ [19] | CH and  DE-NL | 2010  (extrapol. from 2002-2004) | **EUR 9 578  per patient** | **17 642  per patient** | **2 033** |
| Direct health care cost |  |  |  | EUR 7 161  per patient | 13 004  per patient | 1 499 |
| Direct non-medical costs |  |  |  | EUR 2 417  per patient | 4 638  per patient | 534 |
| Total direct cost of stroke (ICD-10 code I61, I63, I64, I67)^e^ | Luengo-Fernandez et al. 2020 [20] | CH and FR-DE-NL | 2002-2017 | **EUR 1 594** | **2 701** | **1 812** |
| Health care cost of disease |  | CH | 2017 | EUR 558 | 942 | 970 |
| Medications |  | CH and FR-DE-NL | 2017 | EUR 33 | 52 | 54 |
| Primary & Outpatient care |  | CH | 2017 | EUR 36 | 62 | 64 |
| Emergency and hospital care |  | CH | 2017 (extrapol. from 2002-2012) | EUR 490 | 829 | 854 |
| Social Care |  | CH | 2017 | EUR 307 | 518 | 534 |
| Informal care |  | CH | 2017 | EUR 170 | 298 | 306 |
| a: Source reported per patient costs instead of overall disease costs  b: Hospitalized patient number reported by Wieser et al 2012 include patients with NSTEMI (ICD-10-I21.4; I21.9; I22.9) and STEMI (ICD-10-I21.0; I21.1–3; I22.0–1; I22.8) in Switzerland 2008 [14]. Patients with unstable angina pectoris (ICD-10-I20) were not used in analysis.  c: Population adjustment for CHD was calculated based on the increased incidence of MI in Switzerland between 2008 (12.785 events) and 2021 (18.966 events) [26]  d: Total = direct healthcare costs & direct non-medical costs  e: Total = healthcare cost of disease, social care & informal care | | | | | | |

Supplementary Table 6: Attributable fraction of overweight and obesity of the direct costs of seven selected overweight and obesity related complications for Switzerland 2021, *based on the annual total changes of healthcare expenditures*

| Disease | Direct cost of illness 2021  [Mio. CHF]^a^ | PAF [ %] | | PAF based costs 2021 [Mio. CHF] | | |
| --- | --- | --- | --- | --- | --- | --- |
|  |  | **Overweight 25 ≤ BMI < 30** | **Obesity BMI ≥ 30** | **Overweight 25 ≤ BMI < 30** | **Obesity BMI ≥ 30** | **Total Attributable direct costs  BMI ≥ 25** |
| Asthma | 2 500 based on [13] | 6.2 | 6.4 | 154 | 160 | **314** |
|  |  | 20.3 | 18.7 | 507 | 468 | **974** |
| CHD | 2 005 – 3 334 based on [14, 15] | 6.6 | 4.7 | 132 – 220 | 94 – 157 | **227 – 377** |
|  |  | 14.1 | 11.3 | 283 – 470 | 227 – 377 | **509 – 847** |
| Depression | 11 078 based on [16] | 3.7 | 5.4 | 405 | 595 | **1 000** |
| Diabetes mellitus | 2 052 based on [17] | 16.2 | 42.9 | 333 | 881 | **1 214** |
|  |  | 38.4 | 41.4 | 788 | 849 | **1 637** |
| Hypertension | 1 657 based on [15] | 18.1 | 16.8 | 299 | 278 | **578** |
| Osteoarthritis (hip & knee) | 3 048 based on [18] | 19.4 ^b^ | 19.6 ^b^ | 591 | 597 | **1 189** |
|  |  | 24.4 ^c^ | 25.0 ^c^ | 744 | 762 | **1 506** |
| Stroke | 2 110 – 3 099 based on [19, 20] | 5.9 | 5.4 | 125 – 184 | 114 – 167 | **239 – 351** |
|  |  | 18.3 | 17.4 | 385 – 566 | 367 – 539 | **752 – 1 105** |
| Overall | | | | **2 039 – 3 779** | **2 719 – 3 868** | 4 761 – 7 647 |
| a: Extrapolation of costs for Switzerland 2021 by conversion with PPP and extrapolation with annual total changes of healthcare expenditures  b: combined PAF based on relative risks for hip osteoarthritis from Holliday et al 2010 and knee osteoarthritis from Muthuri et al. 2011 [9, 10]  c: combined PAF based on relative risks for hip osteoarthritis and knee osteoarthritis both from Lohmander et al. 2008 [8] | | | | | | |

Supplementary Table 7: Total costs of treatment of overweight and obesity as well as direct costs of obesity-linked selected overweight and obesity related complications, *based on the annual total changes of healthcare expenditures*

| Type of costs | Costs [Mio. CHF] |
| --- | --- |
| Direct costs of treatment overweight and obesity | |
| Dietary counselling costs^a^ | 18 |
| Pharmacological therapy costs^b^ | 26 |
| Bariatric surgical therapy costs^c^ | 92 |
| Total direct costs of treatment overweight and obesity | **135** |
| Direct costs of overweight and obesity related complications^d^ | |
| Attributable total direct costs | **4.761-7.647** |
| Total costs of overweight and obesity | **4.897-7.783** |
| a: Costs for dietary counselling were estimated by the most current statistical information of Jan-Jun 2022.  b: Cost for pharmacological therapy costs were estimated by the most current sales information of Jan-Jun 2022.  c: Cost for bariatric surgical therapy costs were estimated based on the number of procedures performed in 2019, as the COVID‑19 pandemic led to a significant reduction in elective surgical procedures performed in 2021.  d: Analysed comorbidities were asthma, CHD, depression, diabetes mellitus, hypertension, osteoarthritis (hip & knee) and stroke. | |

Supplementary Table 8: Search string MEDLINE for cost of selected concomitant diseases


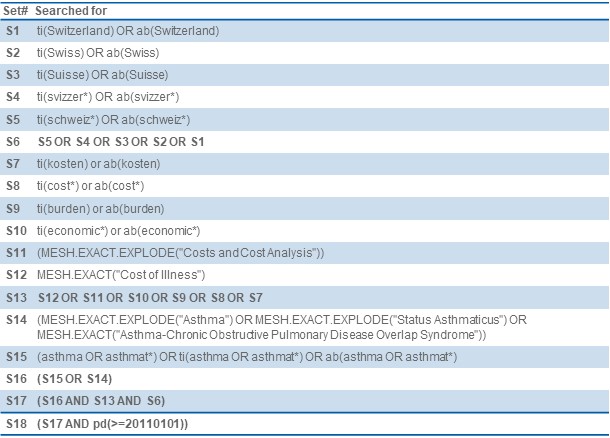


The search was performed on 14^th^ September 2022. Steps „S14 and S15“ contain the disease specific component, which was adapted for each of the selected concomitant treatments as followed:


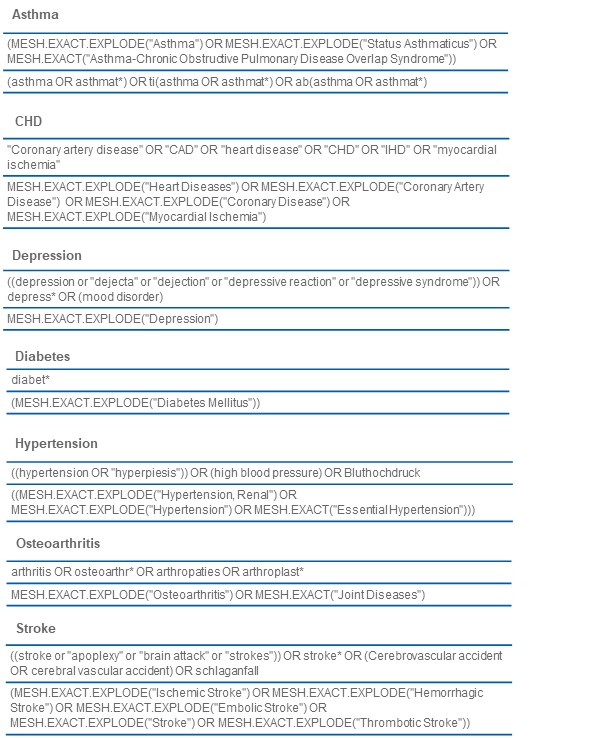


Supplementary Table 9: Search string MEDLINE for Relative risks / PAFs


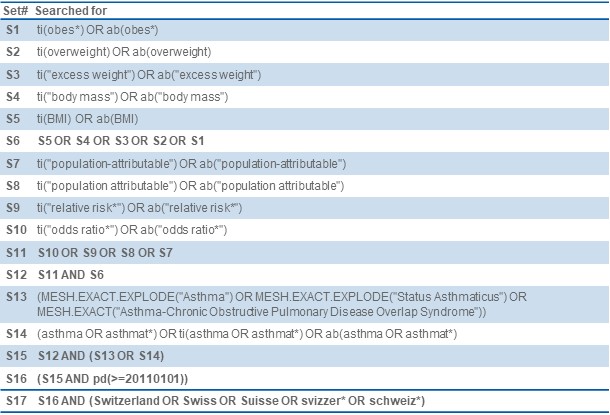


The search was performed on 14^th^ September 2022. Steps „S13“ and „S14“ contain the disease specific component, which was adapted for each of the selected concomitant treatments as described above

**References**

1. Zemp Stutz, E., Ferrari, G., Schneidera, C. Quantitativer Einfluss verhaltensbezogener Risikofaktoren auf das Auftreten nichtübertragbarer Krankheiten. Zusammenstellung der Evidenzen [online]. Stand: März 2020. URL: <https://www.bag.admin.ch/dam/bag/de/dokumente/npp/forschungsberichte/forschungsberichte-ncd/einfluss-risikofaktoren-ncd.pdf.download.pdf/NCD-Bericht_2020-03-06.pdf> [Zugriff: 14.10.2022]. 2020.

2. Ma, J., Xiao, L., Knowles, S. B. Obesity, insulin resistance and the prevalence of atopy and asthma in US adults. Allergy 2010; 65(11): 1455-63. DOI: 10.1111/j.1398-9995.2010.02402.x

3. Flint, A. J., Hu, F. B., Glynn, R. J., Caspard, H., Manson, J. E. et al. Excess Weight and the Risk of Incident Coronary Heart Disease Among Men and Women. Obesity 2010; 18(2): 377-383. DOI: 10.1038/oby.2009.223

4. Liu, L., Núṅez, A. E., An, Y., Liu, H., Chen, M. et al. Burden of Cardiovascular Disease among Multi-Racial and Ethnic Populations in the United States: an Update from the National Health Interview Surveys. Front Cardiovasc Med 2014; 1: 8. DOI: 10.3389/fcvm.2014.00008

5. Luppino, F. S., de Wit, L. M., Bouvy, P. F., Stijnen, T., Cuijpers, P. et al. Overweight, obesity, and depression: a systematic review and meta-analysis of longitudinal studies. Arch Gen Psychiatry 2010; 67(3): 220-9. DOI: 10.1001/archgenpsychiatry.2010.2

6. Davin, C., Vollenweider, P., Waeber, G., Paccaud, F., Marques-Vidal, P. Cardiovascular risk factors attributable to obesity and overweight in Switzerland. Nutr Metab Cardiovasc Dis 2012; 22(11): 952-8. DOI: 10.1016/j.numecd.2011.01.004

7. Guh, D. P., Zhang, W., Bansback, N., Amarsi, Z., Birmingham, C. L. et al. The incidence of co-morbidities related to obesity and overweight: A systematic review and meta-analysis. BMC Public Health 2009; 9(1): 88. DOI: 10.1186/1471-2458-9-88

8. Lohmander, L. S., Gerhardsson de Verdier, M., Rollof, J., Nilsson, P. M., Engström, G. Incidence of severe knee and hip osteoarthritis in relation to different measures of body mass: a population-based prospective cohort study. Ann Rheum Dis 2009; 68(4): 490-6. DOI: 10.1136/ard.2008.089748

9. Holliday, K. L., McWilliams, D. F., Maciewicz, R. A., Muir, K. R., Zhang, W. et al. Lifetime body mass index, other anthropometric measures of obesity and risk of knee or hip osteoarthritis in the GOAL case-control study. Osteoarthritis and Cartilage 2011; 19(1): 37-43. DOI: 10.1016/j.joca.2010.10.014

10. Muthuri, S. G., Hui, M., Doherty, M., Zhang, W. What if we prevent obesity? Risk reduction in knee osteoarthritis estimated through a meta-analysis of observational studies. Arthritis Care & Research 2011; 63(7): 982-990. DOI: 10.1002/acr.20464

11. Winter, Y., Rohrmann, S., Linseisen, J., Lanczik, O., Ringleb, P. A. et al. Contribution of obesity and abdominal fat mass to risk of stroke and transient ischemic attacks. Stroke 2008; 39(12): 3145-51. DOI: 10.1161/strokeaha.108.523001

12. Schneider, H., Venetz, W. Cost of Obesity in Switzerland in 2012 [online]. Stand: 16.06.2014. URL: <https://www.bag.admin.ch/dam/bag/fr/dokumente/npp/forschungsberichte/forschungsberichte-e-und-b/cost-of-obesity.pdf.download.pdf/cost-of-obesity.pdf> [Zugriff: 14.10.2022]. 2014.

13. Szucs, T. D., Anderhub, H., Rutishauser, M. The economic burden of asthma: direct and indirect costs in Switzerland. Eur Respir J 1999; 13(2): 281-6. DOI: 10.1034/j.1399-3003.1999.13b10.x

14. Wieser, S., Rüthemann, I., De Boni, S. N., Eichler, K., Pletscher, M. et al. Cost of acute coronary syndrome in Switzerland in 2008. Swiss Medical Weekly 2012; 142(w13655). DOI: 10.21256/zhaw-3954

15. Destatis. Krankheitskosten, Krankheitskosten je Einwohner: Deutschland, Jahre, Krankheitsdiagnosen (ICD-10) - Krankheiten des Kreislaufsystems [online]. URL: <https://www-genesis.destatis.de/genesis/online?sequenz=tabelleErgebnis&selectionname=23631-0001&sachmerkmal=ICD10Y&sachschluessel=ICD10-I00-I99,ICD10-I10-I15,ICD10-I20-I25,ICD10-I21,ICD10-I22,Icd10-I30-I52,ICD10-I50,ICD10-I60-I69,ICD10-I60-I61,ICD10-I63,ICD10-I64,ICD10-I69,ICD10-I80-I89,ICD10-I83#abreadcrumb> [Zugriff: 14.10.2022]. 2022.

16. Tomonaga, Y., Haettenschwiler, J., Hatzinger, M., Holsboer-Trachsler, E., Rufer, M. et al. The economic burden of depression in Switzerland. Pharmacoeconomics 2013; 31(3): 237-50. DOI: 10.1007/s40273-013-0026-9

17. Huber, C. A., Schwenkglenks, M., Rapold, R., Reich, O. Epidemiology and costs of diabetes mellitus in Switzerland: an analysis of health care claims data, 2006 and 2011. BMC Endocr Disord 2014; 14: 44. DOI: 10.1186/1472-6823-14-44

18. Destatis. Krankheitskosten, Krankheitskosten je Einwohner: Deutschland, Jahre, Krankheitsdiagnosen (ICD-10) - Alle Krankheiten und Folgen äußerer Ursachen [online]. URL: <https://www-genesis.destatis.de/genesis/online?operation=table&code=23631-0001&bypass=true&levelindex=0&levelid=1665749890144#abreadcrumb> [Zugriff: 14.10.2022]. 2022.

19. Maercker, A., Perkonigg, A., Preisig, M., Schaller, K., Weller, M. The costs of disorders of the brain in Switzerland: an update from the European Brain Council Study for 2010. Swiss Med Wkly 2013; 143: w13751. DOI: 10.4414/smw.2013.13751

20. Luengo-Fernandez, R., Violato, M., Candio, P., Leal, J. Economic burden of stroke across Europe: A population-based cost analysis. Eur Stroke J 2020; 5(1): 17-25. DOI: 10.1177/2396987319883160

21. SwissDRG. Datenspiegel 11.0 - Plausibilisierte Leistungs- und Kostendaten der Netzwerkspitäler aus dem Jahr 2019 [online]. URL: <https://datenspiegel110.swissdrg.org/> [Zugriff: 14.10.2022]. 2019.

22. Schweizerischer Verband diplomierter ErnährungsberaterInnen (SVERB), Medizintarif-Kommission-UVG (MTK), Bundesamt für Militärversicherung (BMV). Tarif Anhang A [online]. URL: <https://www.mtk-ctm.ch/fileadmin/user_upload/tarife/Ernaehrungsberatung/Ernaehrungsberatung_ambulant/01_deutsch/ambulante_tarife_ernaehrungsberatung_Tarif_deu.pdf> [Zugriff: 14.10.2022]. 1999.

23. Die Spitäler der Schweiz (H+), Medizinaltarif-Kommission UVG (MTK), Bundesamt für Sozialversicherung (BSV), Bundesamt für Militärversicherung (BAMV). Tarif Anhang 1 [online]. URL: <https://www.hplus.ch/fileadmin/hplus.ch/public/Tarife/Ernaehrungsberatung/Tarif_1_.pdf> [Zugriff: 14.10.2022]. 2002.

24. Schweizerischer Verband diplomierter ErnährungsberaterInnen (SVERB), Medizintarif-Kommission-UVG (MTK), Bundesamt für Militärversicherung (BMV). Vereinbarung über den Taxpunktwert [online]. URL: <https://www.mtk-ctm.ch/fileadmin/user_upload/tarife/Ernaehrungsberatung/Ernaehrungsberatung_ambulant/01_deutsch/ambulante_tarife_ernaehrungsberatung_TPW_deu.pdf> [Zugriff: 14.10.2022]. 1999.

25. Die Spitäler der Schweiz (H+), Medizinaltarif-Kommission UVG (MTK), Bundesamt für Sozialversicherung (BSV), Bundesamt für Militärversicherung (BAMV). Vereinbarung übr den Taxpunktwert [online]. URL: <https://www.mtk-ctm.ch/fileadmin/user_upload/tarife/Ernaehrungsberatung/Ernaehrungsberatung_im_Spital/01_deutsch/ambulante_tarife_paramed_ernaehrungsberatung_spital_TPW_deu.pdf> [Zugriff: 14.10.2022]. 2002.

26. Schweizerisches Gesundheitsobservatorium (Obsan). Indikator Myokardinfarkt [online]. URL: <https://ind.obsan.admin.ch/indicator/obsan/myokardinfarkt> [Zugriff: 14.10.2022]. 2022.
